# Government supervision on quality of smoking-cessation counselling in midwifery practices: a qualitative exploration

**DOI:** 10.1186/s12913-017-2198-z

**Published:** 2017-04-13

**Authors:** Sandra F. Oude Wesselink, Annemiek Stoopendaal, Vicki Erasmus, Déan Smits, Johan P. Mackenbach, Hester F. Lingsma, Paul B. M. Robben

**Affiliations:** 1 Department of Public Health, Erasmus University Medical Centre, Rotterdam, The Netherlands; 2 Institute of Health Policy & Management, Erasmus University, Rotterdam, The Netherlands; 3 Dutch Health Care Inspectorate, Utrecht, The Netherlands

**Keywords:** Government supervision, Inspectorate, Guidelines, Quality of care, Midwives, Smoking cessation, Qualitative study

## Abstract

**Background:**

The Dutch Healthcare Inspectorate supervises care providers in order to improve quality of care. Recently the inspectorate assessed and promoted the use of a guideline on smoking-cessation counselling in midwifery practices. The supervision programme consisted of an announcement of the enforcement deadline for the guideline and site visits. The purpose of our qualitative study was to identify factors related to guideline adherence after the supervision programme, and investigate whether the programme had helped improve adherence.

**Methods:**

We conducted semi-structured interviews with inspected and non-inspected midwives. Additionally, we studied documents and observed the inspection process. The sampled midwives all work in primary care midwifery practices providing care to pregnant smokers. The questions included the current provision of smoking-cessation counselling, support to the midwife in counselling, recent changes in provision of counselling, reasons for recent changes, knowledge about the supervision programme, and experiences with supervision by the inspectorate.

**Results:**

Our results show that guideline adherence depends on several factors. Awareness and familiarity with the guideline are important, as is outcome expectancy. Additionally, motivation, guideline factors and environment factors were mentioned. Besides these previously documented factors, we found that professional collaboration also determined guideline adherence. Increased collaboration in counselling is associated with greater adherence to the guideline, such as provision of counselling and taking required training. The supervision programme helped improve stop-smoking counselling, by making midwives aware of the counselling and giving them an extrinsic motivation to provide counselling.

**Conclusion:**

Motivation and environmental aspects were the most important factors related to guideline adherence, and professional environment was added as significant factor. The improved guideline adherence is partly attributable to the supervision programme.

**Electronic supplementary material:**

The online version of this article (doi:10.1186/s12913-017-2198-z) contains supplementary material, which is available to authorized users.

## Background

Six percent of women in the Netherlands smoke during pregnancy [1]. Among lower educated women, the prevalence of smoking during pregnancy is around 14%. Maternal smoking is associated with a higher risk of foetal mortality and of adverse birth outcomes such as stillbirth, preterm birth, small for gestational age, intrauterine growth restriction, and congenital heart defects [2].

Improvement of quality of care is an ongoing multidimensional process in which various approaches play a role. One approach is external assessment, based on models of peer review, accreditation, and inspection [3]. This study focuses on the inspections enforced under national or regional statutes, whose standards are derived from regulation and existing guidelines [3]. Inspectorates can use various instruments, such as site visits and performance indicators [4]. The main focus lies on the competence of professional staff, compliance with professional standards, and outcomes for service users [4]. In the Netherlands, healthcare supervision is delegated to the national Dutch Healthcare Inspectorate (later: inspectorate) (Additional file 1).

In 2010, the inspectorate began a supervision programme on primary care midwives providing care to pregnant smokers. It focused on the evidence-based Minimal Invention Strategy for Smoking-Cessation Counselling for Midwifery Practices (Minimale Interventiestrategie Stoppen met Roken voor de Verloskundigenpraktijk, V-MIS) [5] (see Context paragraph). The professional guideline recommends providing smoking-cessation counselling to pregnant smokers [6]. Apart from V-MIS, almost no other methods to provide counselling are used. In the period 2010–2012, the inspectorate promoted the use of V-MIS in a supervision programme intended to improve the quality of counselling and reduce smoking rates during pregnancy. The inspectorate collaborated with the Royal Dutch Organisation of Midwives (Koninklijke Nederlandse Organisatie van Verloskundigen, KNOV) and the Netherlands Expertise centre for Tobacco Control (Stichting Volksgezondheid en Roken, STIVORO). In a previous study we found that use of V-MIS increased substantially from 28% in 2010 to 80% in 2012 [7]. This spectacular improvement in adherence to the guideline on smoking-cessation counselling might not be fully attributable to the supervision programme, because other organisations were also involved in promoting quit-smoking counselling. Therefore, we wanted to understand how this improvement was achieved. The purpose of our study was to identify factors related to guideline adherence after the supervision programme, and to investigate whether the supervision programme had helped improve adherence.

## Context

### Minimal invention strategy for smoking-cessation counselling for midwifery practices (V-MIS)

V-MIS comprises seven steps. In step 1, the midwife identifies the smoking behaviour of the woman and partner. In step 2, the midwife attempts to enhance the motivation to quit. In step 3, the midwife and woman discuss barriers for successful quitting and how to mobilise social support for quitting. In step 4, the midwife and woman agree on a quit date. In step 5, the midwives discuss and provide additional self-help materials. In step 6, the midwife provides aftercare if necessary. In step 7, the midwife supports the woman to prevent relapse after delivery. These steps can be provided in one or more consultations. When V-MIS is applied, 12% of the pregnant smokers quit, whereas 3% in the control group quit [5].

### Case: programme of the Dutch healthcare inspectorate

The Dutch Healthcare Inspectorate programme aimed to improve the provision of smoking-cessation counselling to pregnant women by all primary care midwives in the Netherlands.

In 2010, inspectors visited a small sample (10 of 500) of midwifery practices to discuss counselling based on V-MIS with the midwives, first mailing an announcement of the impending visit and the supervision topics. In this exploratory phase, the inspectorate did not enforce compliance to the guideline. Two inspectors visited each site for 2 h, with smoking-cessation counselling as the only topic of discussion. Despite the availability of V-MIS and the guideline, only a minority of Dutch midwives provided smoking-cessation counselling in 2010 [8]. As the inspectorate is supposed to promote public health, part of their job is to monitor and encourage guideline adherence. Therefore, after these preliminary site visits, the inspectorate decided in consultation with the professional organisation to oblige midwives to use V-MIS, because this method is used most frequently and is most suitable for midwives. Then they announced the enforcement deadline of the guideline to all midwifery practices and all 10 inspected practices received a personal report with feedback on their counselling.

In 2012, the inspectorate again visited a sample (21 of 500) of midwifery practices to check whether midwives were complying with the guideline. They inspected policy documents, training certificates and registration forms, and evaluated the use of V-MIS and the midwives’ knowledge of places they could refer women to for support on stopping smoking. Again, mails announced the site visits and supervision topics. Two inspectors took a half day to inspect each practice, spending 10% of the site visit on smoking-cessation counselling and using the rest of the time to address other topics relevant to the quality of midwifery care. Following the site visits, all the inspected practices received a personal report with feedback on their counselling and a time frame for implementing the required improvements. All reports, including the personal reports are available to the public.

The inspectorate’s ultimate measure is to shut down a midwifery practice, in which case that practice cannot accept new clients and must hand over current clients to other midwifery practices. The inspectorate has never applied this ultimate measure to any Dutch midwifery practices, but does so occasionally in nursing homes, home care organisations and hospital departments [9].

Alongside the inspectorate, healthcare insurance companies may audit guideline adherence. Insurers may ask practices for improvements to the quality of specific aspects of care. The insurers’ ultimate measure is to cancel their contract with a midwifery practice so that the midwives receive no payment for clients insured by that insurance company.

### Support for midwifery practices on smoking-cessation counselling

The aim of the Netherlands Expertise centre for Tobacco Control (STIVORO) is to promote a cigarette smoke-free future. The professional midwifery organisation strives at the best care for pregnant women and their partner. STIVORO and professional midwifery organisation collaborated in the provision of support to midwifery practices to improve smoking-cessation counselling.

During the supervision period, the facilitation of smoking-cessation counselling improved. Both STIVORO and the professional organisation committed to helping midwifery practices improve counselling, after a consultation with the inspectorate. In 2011, STIVORO discovered that fewer midwives were taking training courses, although this was very important for improving counselling. Redistributing its funds, STIVORO then arranged a discount for the training course and announced this through various channels facilitated by the professional midwifery organisation. The discount made the training very attractive to midwives. In its communication, STIVORO mentioned the enforcement by the inspectorate. The course also paid attention to other referral options that would support pregnant smokers. Besides collaborating on the training course, STIVORO and the professional midwifery organisation jointly published a handbook on smoking-cessation counselling [10]. The midwifery practices could use this handbook to formulate their policy on smoking-cessation counselling in their practice. Lastly, STIVORO requested the software companies who provide software for patient record systems to include items on smoking-cessation counselling in the electronic patient record. Based on V-MIS, the items include the preferences of the pregnant smoker and the actual care provided by the midwife. Such enhancements improved the continuity of smoking-cessation counselling. For more details on perinatal care in the Netherlands and smoking-cessation counselling see Additional file 2.

## Theoretical framework

To identify factors related to guideline adherence and investigate the contribution of the inspectorate, we applied two different theories. The behaviour of midwives we describe with Cabana’s guideline framework [11] and the behaviour of the inspectorate according to the responsive regulation theory [12].

Guideline adherence is determined by various factors. The sequence of behaviour change ranges from knowledge through attitudes to behaviour [11]. For knowledge, it is important to be aware of and familiar with the guideline. This includes, for example, the amount of information, the time needed to stay informed, and guideline accessibility. Attitude is determined by several factors including agreement with specific guideline characteristics, agreement with guidelines in general, outcome expectancy, self-efficacy, and motivation. Outcome expectancy refers to whether the midwives believe that following the guideline recommendations will lead to the desired outcome, in our case that pregnant women quit smoking. Self-efficacy means that the midwife believes that they can follow the guideline recommendations. Lastly, behaviour is influenced by external barriers, guideline factors, and environmental factors, which include time, resources, organisational opportunities, and reimbursement.

The inspectorates stimulates guideline adherence through responsive regulation. This method of supervision uses the reactions of the regulated entities to determine the degree of supervision, applying an enforcement pyramid, which ranges from persuasion at the bottom to license revocation at the top [12]. The idea behind the pyramid is that it will be easier to persuade regulated entities to follow the guidelines if they know about the ‘big guns’ (deterrents). In this case the deterrent is the power of the inspectorate to close the midwifery practice. The pyramid also shows that for small violations that care providers are willing to improve, the inspectorate has to start with the lowest step of the pyramid and not with the big guns.

## Methods

### Data collection

Midwifery practices were first approached by e-mail and later by phone. Interviews took place at the midwife’s workplace and were conducted preferably with the midwife responsible for smoking-cessation counselling in the practice. The interviewer and midwife had no pre-existing relation. All interviews lasted between 30 and 60 min and took place between March and June 2013.

One researcher (DS), an MSc student trained and experienced in conducting interviews, did all the interviews.

Besides the interviews, we collected additional data from the supervision programme. We observed meetings of inspectors and inspections and, to be as well informed as possible, collected minutes and other documents by the inspectorate. During these observations, the researchers always introduced themselves before the observations. The details of the study, including the fact that the inspectorate initiated this research, were explained.

### Study population

The study population was selected using purposive sampling (REF Patton 2002), resulting in three groups of midwives working in primary care midwifery practices. The first group contained midwifery practices that were inspected in 2010. We approached 8 practices of which 5 took part in this study. The second group consisted of midwifery practices that were inspected in 2012. We approached 7 practices of which 4 participated. The third group held a random selection of practices that had not been inspected. Here we approached 11 practices, of which 5 agreed to participate. Reasons for refusal were lack of time and not interested in participating since the practice was not inspected. All practices were selected randomly and practices with any previous involvement in our research were excluded. This ensured that all practices included in the current study were not included in other studies performed by the Department of Public Health, Erasmus Medical Centre. As we used purposive sampling, we did not check for data saturation.

### Interview guide

The interviews were based on an interview guide (Table 1). The questions were develop by the researchers using recent literature on the effect of inspections. The questions were about the current provision of smoking-cessation counselling, support to the midwife in counselling, recent changes in provision of counselling, reasons for recent changes, knowledge of the supervision programme, and experiences with supervision. The interview questions were first tested by the interviewer (DS) on 2 researchers.

**Table 1:** Interview guide

Smoking-cessation counselling
1. How do you provide smoking-cessation counselling to pregnant smokers? What did you change in care to pregnant smokers last years? Which support can you turn to?
2. What did your colleagues change in care provided to pregnant smokers?
3. Which support did you receive in the care for pregnant smokers? Is this changed last years?
Inspection
4. What did you hear about the supervision programme on midwife practices with respect to smoking-cessation counselling? How have you obtained this information?
5. Why conducted the inspectorate this supervision programme according to you?
6. Have you read the supervision report or the publication in the journal of your professional organisation?
7. To what extent was this publication recognisable to you?
What changed as a result of inspection?
8. To what extent did the inspectorate contribute to this change? - Were these changes affected by other actors?
9. To what extent did the inspectorate contribute to the change of your colleagues?
Why did you change the way you work?
10. Which aspects contributed to compliance to instructions from the inspectorate? Conceptual model: autonomy, workload, way of inspection, motivation, field standards, transparency, trust, (in)dependence of the inspectorate, expectations and relationship with the inspectorate
Change in inspection
11. If you should perform the supervision, how would do it? How would be the impact of the supervision on your work be the largest?

### Data analyses

All interviews were audio recorded and fully transcribed. Names and privacy-related information were removed. Interview transcripts underwent systematic content analysis based on grounded theory [13], using NVivo software, version 10 (QSR international, Doncaster, Australia). Phrases were combined to generate categories. This process continued until all transcripts were analysed and no new categories emerged. Subsequently, the content of the categories was analysed for overlapping or linking content. All this was done by one researcher (SOW). The categories were then compressed and clustered into themes. The themes were evaluated across the different groups and respondents to search for similarities and differences. Finally, we analysed the data using Cabana’s model [11] and clustered the information into the guideline adherence factors. This was done by two researchers (AS and SOW).

Information from observations and document analyses were used as background information to understand the outcomes of the interviews. Therefore, they were not transcribed. A previous quantitative study on supervision on smoking-cessation counselling also functioned as background information [7].

## Results

### Population

In total 14 midwives participated in our study (Table 2). The average age of midwives was 44 years in the inspected group and 45 years in the non-inspected group. Almost all midwives were female, except one in the non-inspected group. In both groups, one midwife smoked and in the inspected group, two midwives were former smokers. Four midwives were physically present during the inspections of the inspectorate. On average, participants in the inspected group worked 16 years as a midwife and 11 years in this practice. Participants in the non-inspected group worked 18 years as a midwife and 15 years in this practice. The inspected group treated on average 28 smokers in their practice per year and the non-inspected group 48 smokers per year. The interviews lasted on average about 44 min in the inspected group and 40 min in the non-inspected group.

**Table 2:** Characteristics of participants (*n =* 14)

	Inspected midwives (*n =* 9)	Non-inspected midwives (*n =* 5)
Age in years (mean)	44	45
Female	9	4
Smoking behaviour midwife:	6	3
Smoker	1	1
Past smoker	2	0
Present during inspection	4	
Years working as midwife (mean)	16	18
Years working in this practice (mean)	11	15
Duration interview in minutes (mean)	44	40

### Awareness and perceived effectiveness of the guideline and supervision programme

All midwives in both inspected and non-inspected midwifery practices were informed about the supervision on smoking-cessation counselling. This information reached them through various channels. Some practices indicated that they received the information directly from the inspectorate by letter or report. Others stated that the professional organisation told them about the supervision. The last possibility was that practices were informed through neighbouring practices that had received an inspection.

*“I can remember it. I got a letter. It was a few years ago. (…) So, the letter did get here.” Midwife, female, not inspected by inspectorate*

All midwives knew about the existence of the supervision on smoking-cessation counselling and were thus informed about the guideline. The midwives said that the supervision programme showed them how to improve their counselling. The midwives felt that counselling was effective: pregnant smokers smoked less or counselling did not take much extra time. Midwives are against smoking and are strongly motivated to strive for the good health of mother and child. Midwives are committed to healthy pregnancy and delivering a healthy baby. Perinatal audits raised awareness of the harm of smoking. In these audits, midwives in the multidisciplinary obstetric partnership discuss all the babies who had died during pregnancy or delivery. In almost all cases, the baby’s mother was a smoker.

Some inspected practices were rated as ‘inadequate’ by the inspectorate. The midwives indicated that this rating caused embarrassment and disappointment in the practice. The public availability of the report enhanced these feelings. However, in these practices it also worked as an extra motivation to provide better counselling. Further, the public reporting created extra awareness in other midwives who were not inspected by the inspectorate.

### Familiarity with guideline

Care providers are deemed familiar with a guideline when they can correctly answer questions on the guideline and when they self-report familiarity. Midwives wanted to improve their familiarity with the guideline and smoking-cessation counselling:

*“I felt that my counselling to pregnant smokers was not good enough. I wanted to learn more about how to provide neutral and effective counselling.” Midwife, female, inspected by inspectorate*

The midwives gained familiarity during courses in stop-smoking counselling which also contained information on external referral possibilities:

*“During the course we learned how we should or can refer people, to let them quit smoking. It’s the task of the GP, but we need to refer them.” Midwife, female, inspected by inspectorate*

They also learned how to refer from colleagues or through information they collected themselves:

*“We have a map in this tray that shows where we send them to (referrals), the outpatient clinics and so on. So it’s become easier and clearer.” Midwife, female, inspected by inspectorate*

These improvements made it easier to refer pregnant smokers to organisations outside the midwifery practice. This familiarity improved guideline adherence. However, despite a range of improvements, in some regions referrals are still not optimal. The guideline provides no clear guidance on how to inform midwives about external referral options or how often midwives require training in counselling.

### Outcome expectancy

Guideline adherence should in principle lead to the better outcomes. However, there may be also unintended outcomes. Some practices indicated to be less strict in providing smoking-cessation counselling because clients left the practice after the midwife tried to persuade them to quit smoking:

*“I work on a small scale. We always want to keep our clients. When I come down hard on a pregnant smoker’s behaviour and the next practice doesn’t do that, and my client hears about it, she can easily switch practices. No midwife wants that. (…) It has happened. One left my practice.” Midwife, female, inspected by inspectorate, stopped smoking 18 months ago*

Many practices reinforced their counselling after inspection. An unintended side-effect was that some clients left the practice, which had a huge impact on the midwives. Midwives said that they do not want to be known as more rigorous than other practices. Reputation is very important for them given that a bad reputation can lead to fewer clients registering at their practice and consequently less work and lower income. If a client left the practice, the midwife will decide to ease up on the counselling a little. However, if she followed the minimal intervention strategy carefully, clients should feel supported, not offended, by the midwives. Midwives who struggle with the methods might need more training to provide counselling without upsetting their therapeutic relationship with clients. The training in counselling provision might be too short for midwives who find it harder to provide counselling. Regularly repeating the course might be an option for them. One practice found another solution to improve counselling, without burdening the midwives. Here they referred pregnant smokers to other care providers in the practice. A specialist addiction nurse provides counselling to pregnant smokers, which might be more effective.

If the guideline adherence leads directly to the expected outcome, the situation is totally different:

*“When it’s an improvement, I feel good about it. I believe everything can go to work towards a better outcome. That’s very important for me, a good outcome.” Midwife, female, not inspected by inspectorate*

This midwife indicates that counselling works as an improvement of care. Other midwives report counselling as standard provided care. Some midwives said that they do not feel as if it affects their autonomy, because the care improves. So, they can accept the inspections easily:

*“It’s for a good cause, what they do. They want to improve the quality of care.” Midwife, female, inspected by inspectorate*

There is a common interest in improving quality of care. The midwives said that they understand and respect the inspectorate, although they think the inspections are inconvenient.

### Self-efficacy

The belief that one can actually perform certain behaviour is called self-efficacy. The practices that did not improve their smoking-cessation counselling gave various reasons for this. The midwife’s own smoking behaviour might play a role:

*“My locum also smokes and she almost never talks about it with clients. (…) The point, of course, is since you smoke too, you don’t ask about it at every consultation.” Midwife, female, not inspected by inspectorate, smoker*

It seems hard to advise pregnant smokers to quit, if you yourself are also unable to quit. Both smokers and non-smoking midwives suggested this. On the one hand, one can argue that smoking midwives are connected more to pregnant smokers, but our study showed that it is mostly the other way around; midwives who smoke give almost no counselling. This is a lack of self-efficacy. The midwife does not believe that she can follow the guideline, because she smokes.

Self-efficacy is also needed to complete the training:

*“Interviewer: Did you take any training courses in smoking-cessation counselling?*

*Respondent: No, none. Nothing (…) It just didn’t happen.” Midwife, male, not inspected by inspectorate, non-smoker*

This example shows that not all practices have one midwife trained in smoking-cessation counselling. These practices are thus not following the guideline.

### Motivation

Lack of motivation can hinder guideline adherence in many ways. In one practice the responsible midwife, who is probably also the most motivated, left the practice:

*“She was the specialist, but she left the practice last year and now we the same problem again.” Midwife, female, inspected by inspectorate*

The practice has lost knowledge, because the midwife left. There is also a risk of deterioration in smoking-cessation counselling, because no one has been made responsible. The lack of motivation now hinders guideline adherence.

Motivation can be both intrinsic and extrinsic. This midwife is extrinsically motivated, by the obligation of the guideline:

*“I assume we had to. (…) Because somebody had to do it. And it does motivate, when things are mandatory, than somebody does it.” Midwife, female, not inspected by inspectorate, non-smoker*

The midwives said they felt a sense of duty to the inspectorate and professional organisation. If these organisations told them to do something, they reported, then they would want to follow the instruction. They wanted to adhere to rules and protocols. However, some midwives were intrinsically motivated. They said that wanting to take training courses belongs to their professional attitude.

Lack of motivation can also lead to specific non-adherence. For example, the midwives had to buy (and pay for) the self-help materials themselves. Some midwives said that they ordered these materials together with other practices in the circle, because large orders received more discount. Previously the materials were free, but in recent years the price of leaflets has gone up:

*“I distribute stuff from the outpatient-smoking clinic at the hospital. Before, it came from STIVORO, but this material is no longer free and I don’t understand why I should have to pay for self-help materials for clients.” Midwife, female, inspected by inspectorate, non-smoker*

Less self-help material is distributed among pregnant smokers, because of increasing costs. However, cost is not the *only* important aspect to consider:

*“We don’t have the V-MIS self-help material, because you have to pay to get it. We requested it a while ago, but we had to pay a significant amount for it. (…) I’m willing to inform people for the good of the cause, but why should we care providers have to pay for it?” Midwife, female, inspected by inspectorate, smoker*

Although the provision of counselling is more expensive than the supportive self-help materials, the midwives decided to stop buying them.

Motivation is also important for following the preferred training courses. Some midwives did not bother going on a course in counselling:

*“The range of education available is so broad you have to make choices at a certain moment in time. So you go on courses for just the urgent problems in your practice. It has to do with incidence, and urgency, yes, in your practice.” Midwife, female, inspected by inspectorate, smoker, practice with 20–25 smokers a year*

This midwife did not take the training course, because the staff of the practice had agreed that a colleague would go instead. Other practices reported that their staff did not go to the course because other courses were more important to them. However, there was also extrinsic motivation for those who did go to the course. For example, midwives must follow several hours of training a year to stay in the quality register or because they thought that the course would look good on their CV. In general, most midwives said that they took the counselling course because it was easy to attend as it was put on in their region or during their midwifery education.

Motivation also relates to the perception of the midwife’s task. Midwives differed in their opinion on whether they are responsible for their clients’ addictions. Some midwives stated that they were not responsible and therefore they did not follow the guideline.

In summary, the reasons for following the prescribed training were both intrinsic and extrinsic. Midwives indicated that they wanted to learn more about effective counselling or felt forced by the inspectorate to take the course. The reasons for not taking the training relate to the attitude and motivation of the midwife. Non-attendees see the training as less important, compared to other courses and activities. In some cases this also relates to the number of pregnant smokers in the practice. Non-attendees have relatively few pregnant smokers in their practice.

### Guideline factors

The organisation that prescribes the guideline affects adherence. However, we found that this is not the same for all midwives. Midwives differ in their opinion about the relation between midwives, the inspectorate and the professional organisation. According to the midwives, the professional organisation is closer to the midwives than the inspectorate is. The inspectorate is independent and therefore at a greater distance. However, this distance is interpreted differently by different midwives. Some find the advice of the professional organisation more important:

*“I think that the professional organisation is more credible for me. (…) Because they are there for the midwives. (…) If the professional organisation says, ‘You shouldn’t use this programme’ and the inspectorate says ‘No, you must use it’, then I’d say, let’s use the standard programme and follow the advice of the professional organisation.” Midwife, female, inspected by inspectorate*

The professional organisation defends the interests of the midwives and is therefore experienced as a leader. For other midwives this close collaboration leads to less pressure:

*“I think a letter from the inspectorate comes across stronger than a letter from the professional organisation, because we have more correspondence with the professional organisation, and less from the inspectorate.” Midwife, female, inspected by inspectorate*

Here the midwife says that a letter from the inspectorate has more influence in the practice, than one from the professional organisation, merely because the inspectorate is at more of a distance. Trust in the professional organisation and inspectorate also differs between practices. Some practices have more trust in the inspectorate, as an independent organisation. Others have more trust in the professional organisation, because they represent the interests of the midwives:

*“I do trust the inspectorate, but the professional organisation is for our profession, so I trust them more.” Midwife, female, not inspected by inspectorate*

As the professional organisation is the advocate of the midwives, the midwives feel more connected to them. The inspectorate is seen as an organisation higher up in hierarchy, which underlines their independence:

*“The inspectorate was decisive for me, because they are another agency. The professional organisation is an association and I can follow what they say or not. The inspectorate is the highest agency in the hierarchy and they have to check whether people in healthcare are providing good care.” Midwife, female, inspected by inspectorate*

The midwife recognises the legal status of the inspectorate. However, many midwives believe the professional organisation is more credible. Some employees of the professional organisation also work in midwifery practices, and not only in desk jobs. This helps them to have a good view on the practices of providing care.

The collaboration of the professional organisation and inspectorate is seen as important:

*“I would prefer that the inspectorate works with the professional organisation. No inspections without the professional organisation, because they are the representatives of all midwives.” Midwife, female, not inspected by inspectorate*

Different midwives think differently about their position in relation to the professional organisation and the inspectorate. As the influence of these institutions on midwives is different, both can benefit from the differences in opinions by collaborating where possible. Close collaboration makes their message stronger and that leads to a coherent message to the midwives.

### Environmental factors

Some factors that inhibit or foster guideline adherence are beyond the control of midwives. During the supervision programme, digital registration of smoking-cessation counselling became available and many practices switched to this new system. They felt it was an improvement as the digital registration works as a reminder and checklist, and it facilitates collaboration and transfer of information within the practice:

*“It’s easy to get at; you don’t have to open other programmes.” Midwife, female, inspected by inspectorate*

Many midwives started digital registration when it became available. One midwife said that digital registration was not possible because the computer crashed when she tried using it.

For external referrals, midwives depend on other organisations. Midwives reported that they had created a ‘social map’, which displays the external organisations to which they can send referrals. An example of referral options is individual coaching by care providers from mental healthcare practices, the GP or a practice nurse. They felt this was an improvement. They referred more clients once they had made a social map. However, the availability of external referral options changes from time to time. Some programmes that provide counselling ended or the coverage of the insurance company changed, which made a programme unattractive for pregnant smokers. This led to an unclear situation whereas keeping a social map up-to-date requires continuous time and effort:

*“It’s not so clear, here. Yes, we always do (refer to the) GP, if necessary, but more than that? I know some regions have special lung clinics for outpatients who do something, but we don’t have that listed here (on our social map).” Midwife, female, not inspected by inspectorate, non-smoker*

This midwife suggests other improvements to help pregnant smokers, but she is unable to arrange them. An important barrier is restricted time. Although midwives who provided counselling stated that it did not cost them much time, the midwives who did not provide counselling indicated that it would cost a lot of time. They believed that other care providers have more time for it.

*“I imagine it will take far more time. We see 400 pregnant women a year. If you spent one hour… Although, not all smoke. (…) It (still) costs a lot of time.” Midwife, male, not inspected by inspectorate, non-smoker*

Despite the fact that the counselling is effective, midwives have to invest extra time in caring for pregnant smokers. That extra time costs money and leaves less time for other important problems. These barriers are related to external factors, such as time and money restrictions. In addition, the influence midwives have on pregnant smokers is limited:

*“We (the Dutch government) banned the cigarette from pubs and restaurants, and that’s great, it’s a huge improvement, but now you still see people smoke outside. (…) If you smoke and if health insurance companies have a say in it. (…) A bonus, simple as that: if you don’t smoke, you get a bonus (discount on your premium) from your insurance company. That’s a good idea, we should do that!” Midwife, female, inspected by inspectorate*

This midwife feels that national policy measures have more effect on smoking than her own efforts and that influences her counselling. She provides counselling, because it is a good way of getting pregnant women to quit smoking. However, some midwives doubt the impact on the national scale.

### Professional collaborations

In addition to the Cabana model [11], we found that professional collaboration is an important factor in guideline adherence. Midwives indicate that it was easy to change the smoking-cessation counselling if the practice changed their composition of midwives. Midwives said that regional collaboration between midwives and in multidisciplinary obstetric partnerships led to improvements in counselling. A colleague’s recommendation was reason enough to take the training course. The agreements made in these collaborations increased the motivation to adhere to these agreements. All practices had to formulate a protocol and sometimes they worked together on the draft protocol. If one practice wrote a protocol, other practices used it as well:

*“We often try to work together and combine our efforts. (…) And if somebody writes a policy document, we can all use it.” Midwife, female, inspected by inspectorate*

Although practices are partly competitors, they try to cooperate where possible. These cooperation practices can save them time and money and they can learn from each other. The practices that provided less counselling had no collaboration in-house or a multidisciplinary obstetric partnership. These midwives said they worked very much as individuals in the practice. Sometimes there was even no collaboration with the professional organisation. In some cases, the multidisciplinary obstetric partnership with the hospital paid no attention to counselling:

*“The gynaecologists just say: we don’t have time for that.” Midwife, female, inspected by inspectorate*

Midwives find it demotivating when the gynaecologists have no time for smoking-cessation counselling. The midwives think that gynaecologists do not see the importance of counselling.

In the previous quotes it is apparent that professional collaboration makes it easier to follow the guideline. Practices do not have to do everything by themselves; collaboration makes guideline adherence efficient and they can avoid delivering lower quality of care than surrounding practices. The professional collaborations also played a role in the decision to take counselling training. Because the professional midwifery organisation and colleagues recommended this course, some midwives actually took it. On the other hand, lack of professional collaboration inhibits guideline adherence. For example, the gynaecologists’ negative attitude, that they have no time for counselling.

The Cabana model [11] also mentions ‘view on guidelines in general’, ‘view on this specific guideline’ and ‘patient factors’ as important factors for guideline adherence. However, our participants did not specifically mention these factors.

### Responsive regulation

The theory of responsive regulation suggests that if the ultimate measure is known, the care provider will be more inclined to follow the guideline [12]. One midwife said she was afraid of the inspectorate inspecting her practice:

*“I was scared that. (…) I thought that you wouldn’t get your money back again and it all was so compulsive.” Midwife, female, not inspected by inspectorate*

Despite the fact that the midwives in our study knew perfectly well about the ultimate measure, not all adhered to the guideline. An explanation for this contrary result might be that in the Netherlands no midwifery practice was ever closed. Although the midwives knew about the power of the inspectorate to close a midwifery practice, none had experienced the ruling put into force.

The guideline was ambiguous about the obligation to follow the guideline. For example, the practices should have a ‘social map’ and be trained in counselling. But the guideline provides no information on the required timeframe for updating the social map or taking training courses. Zuiderent-Jerak [14] described the lack of clarity on the guideline obligations, which can lead to reduced guideline adherence. Care providers will follow the guideline more often if the obligation to do so is clear. This is also related to responsive regulation: supervision is complicated if it is not clear when a rule is violated and the inspectees do not know when a regulator can sue them.

## Discussion

### Summary of main findings

Guideline adherence depends on several factors. In our case study, awareness and familiarity with the guideline and supervision programme were important, as was outcome expectancy. We discussed extensively motivation, guideline factors and environment factors. Besides these previously documented factors, professional collaboration also determined guideline adherence. More collaboration in counselling is associated with more guideline adherence, such as provision of counselling and taking the required training. The supervision programme contributed to improvements in stop-smoking counselling, making midwives aware of the counselling and giving an extrinsic motivation to provide counselling.

### Strengths and limitations

The design of this study has several strengths and limitations. One strength is that our sample of midwives was a mixed group differing in age, work experience, practice situation and whether they smoke. This enabled us to record many opinions on smoking-cessation counselling and inspection. A second strength is that the midwives in our sample were exposed to various kinds of supervision. Some were inspected and others were not, also in different rounds. Therefore we could also describe the potential effects of supervision on uninspected practices. The last strength is that our data collection included the whole supervision programme. This resulted in information on all aspects of the programme.

A limitation of the study is that it is based solely on interviews. We could not check the answers on social desirability because we did not observe the midwives at work or analyse documents. However, we obtained extensive information from the interviews on why the midwives did what they did. In observations and document analysis you can only see what they do and not retrieve any information on the ‘why’. In addition, we interviewed midwives only, and no other health care professionals involved with pregnant women or inspectors. However, besides the interviews, we did collect additional data on the supervision programme, which provided the facts and intentions of the supervision programme, in addition to the experiences of the care providers.

A second limitation is the possibility of recall bias. Some midwives were inspected about three years before the interview took place. This intervening period might have been too long to provide enough insights in motivations of their actions at the time.

The final limitation is that our study contained only one case of supervision. Therefore it is difficult to generalise our results to other supervision programmes. When comparing midwives to general practitioners, we believe that the profession of midwife is closely related to the GP’s. Both care providers are situated in the neighbourhood, close to their patients. Their types of practice are comparable; both private (individual) and with employees are possible. The referral options for smoking-cessation counselling are also comparable. Initial training for GPs is much longer than for midwives, but their post-initial requirement training is comparable (200 h every five years). All this might indicate that our case is transferable to other primary care providers, for example GPs.

### Interpretation

This study followed a previous quantitative study which found that V-MIS use increased substantially from 28% in 2010 to 80% in 2012 [7]. After our current qualitative study, we can conclude that this improvement is related to the supervision programme. However, it is not fully attributable to the supervision programme, since other stakeholders also played roles in the improvement. Our combination of study methods provides additional knowledge on the effectiveness of the supervision programme and the factors that contribute to guideline adherence.

Our study found two groups of midwives who were intrinsically and extrinsically motivated to adhere to the quit-smoking guideline. The intrinsically motivated midwife acts at once when she hears of opportunities to improve counselling. For example, when she hears about training courses, she immediately signs up, whereas an extrinsically motivated midwife first needs an external motivation, such as advice from a professional organisation or the force of the inspectorate. Most midwives want to follow guidelines and the advice from the inspectorate, if they aim at a common goal. Although an extrinsically motivated midwife also follows the guideline, the lack of intrinsic motivation carries a risk. If the external motivation is omitted, the quality of her counselling might deteriorate to the old level.

In our analysis we focused on whether improvements resulted from the supervision programme. Despite this focus, we also encountered deterioration in quality of care caused by, for example, a trained midwife leaving the practice or the counselling was provided less strictly to pregnant smokers. Since the inspectorate took a large part of the responsibility to improve counselling, it could be that midwifery practices feel less responsible for ongoing improvements to counselling. Following the supervision programme, responsibility should be returned to the practices to enhance the self-regulatory capacity of the midwives. Our data collection took place 18 months after the deadline for guideline adherence imposed by the inspectorate. Even in this short time, we noted some deterioration in guideline adherence. As this deterioration began quite soon, it is important to prevent further deterioration in the future.

## Conclusion

Further research should investigate whether the results of our study are generalizable to other supervision practices. Studies with additional participating observations or document analysis in care practices might be useful to obtain more insight and evidence on whether supervision programmes are effective and how they work. Furthermore, the focus can be extended towards deterioration in quality of care after the supervision programme has ended.

In this study we found that the combination of methods used to distribute the supervision programme was successful. Future programmes should also aim at using multiple elements in each supervision programme to reach all targeted care providers.

Most of the factors determining guideline adherence found in this study were in line with Cabana’s model [11]. Additionally, we found that professional collaboration also has an impact on guideline adherence. Therefore, we recommend considering professional collaborations when attempting to improve or measure guideline adherence.

As we also found obstacles that inhibited improvements in counselling, we recommend giving attention to causes of deterioration in quality of care. This attention should not necessarily come from the inspectorate, because care providers are also responsible for improving quality of care. However, the inspectorate can monitor whether the profession is paying attention to this issue.

Both the professional organisation of midwives and the inspectorate are seen as policy makers. Collaboration between them strengthens the message of the importance and requirements of quit-smoking counselling.

In conclusion, we explored factors related to adherence to a guideline on smoking-cessation counselling in midwifery practices. Motivation and environmental aspects were the most important factors related to guideline adherence, and professional environment was added as significant factor for guideline adherence. The improved guideline adherence is partly attributable to the supervision programme.

## Additional files

Additional file 1: Dutch Healthcare Inspectorate. Dutch Healthcare Inspectorate. Brief summary about Dutch Healthcare Inspectorate. (PDF 34 kb)

Additional file 2: Perinatal care in the Netherlands and smoking. Perinatal care in the Netherlands and smoking-cessation counselling. Description of actual situation of perinatal care in the Netherlands and how smoking-cessation counselling for pregnant women is organised. (DOCX 16 kb)

## Abbreviations

STIVORO: Netherlands Expertise centre for Tobacco Control
V-MIS: Minimal Invention Strategy for Smoking-Cessation Counselling for Midwifery Practices
