# Supplementary material for: Government supervision on quality of smoking-cessation counselling in midwifery practices: a qualitative exploration
Source: BMC Health Serv Res. 2017 Apr 13;17:270. doi: 10.1186/s12913-017-2198-z (PMC5390412; doi:10.1186/s12913-017-2198-z)
Supplement: Supplementary file 1 — Dutch Healthcare Inspectorate. Dutch Healthcare Inspectorate. Brief summary about Dutch Healthcare Inspectorate. (PDF 34 kb) [file 12913_2017_2198_MOESM1_ESM.pdf]

## Additional file 1 Dutch Healthcare Inspectorate

The Dutch Healthcare Inspectorate (Netherlands, EU) is an independent agency of the Ministry of Health, Welfare, and Sport. It aims to improve population health and is expected to efficiently supervise a sector where 1.3 million people work for 40,000 institutions and companies. Its primary instruments are advice and encouragement. If these do not achieve the desired result, it can implement corrective action by, for example, increasing the supervision or by limiting the ability to practice a profession. The inspectorate enforces 25 laws, including for example the Care Institutions Quality Act. The supervision is performed by using a combination of three methods:

1. theme-based supervision, directed at specific issues in care, which are sometimes requested by the minister or parliament
2. supervision in response to calamities or emergencies that indicate structural shortcomings in care provision
3. risk-based supervision to assess the quality of healthcare by means of indicators.

The programme described in this study is an example of theme-based regulation.
