# Supplementary material for: Government supervision on quality of smoking-cessation counselling in midwifery practices: a qualitative exploration
Source: BMC Health Serv Res. 2017 Apr 13;17:270. doi: 10.1186/s12913-017-2198-z (PMC5390412; doi:10.1186/s12913-017-2198-z)
Supplement: Supplementary file 2 — Perinatal care in the Netherlands and smoking. Perinatal care in the Netherlands and smoking-cessation counselling. Description of actual situation of perinatal care in the Netherlands and how smoking-cessation counselling for pregnant women is organised. (DOCX 16 kb) [file 12913_2017_2198_MOESM2_ESM.docx]

# Appendix 2 Perinatal care in the Netherlands and smoking-cessation counselling

In the Netherlands, pregnant women have a free choice for place of birth, including at home ([8](#_ENREF_8)). For low-risk pregnancies and deliveries midwives may provide care on their own during gestation, childbirth and the postpartum period. All midwives have obtained a bachelor’s degree from a university of applied sciences ([9](#_ENREF_9)). This education involves four years of theory and internships combined. After graduation, midwives have to take at least 200 hours of training courses and further education every five years to stay listed in the quality register for professional midwives.

Primary care midwives work in private practices, either as self-employed practitioners or as employees in someone else's practice. Self-employed practitioners work alone or in partnership with one or more other midwives. Many midwives work as locums, filling in temporary vacancies in midwifery practices.

Most practices work with teams of 3 to 5 midwives caring for one pregnant woman, with one team member assisting at delivery and the team sharing information on the woman through the electronic patient file. Each practice has a midwife on call 24/7. In the past few years, most hospitals work with multidisciplinary obstetric partnerships, involving all birth care providers in their hospital, including primary and secondary midwives, gynaecologists, paediatricians, maternity nurses and obstetric general practitioners.

Primary care midwives are paid per care unit, separately for pre-natal, natal and post-natal care ([10](#_ENREF_10)). Consequently, the practice suffers a financial loss if a client leaves the practice, especially during pre-natal care. To be paid, practices are required to have contracts with healthcare insurers ([8](#_ENREF_8)). The healthcare insurers may ask for improvements in quality of care when negotiating contracts.

According to the guideline, midwives must provide smoking-cessation counselling to pregnant smokers. A minimal intervention strategy, V-MIS increases the quit-smoking rate in pregnant smokers ([5](#_ENREF_5)). It targets midwifery practices and is based on the stages of change theory ([11](#_ENREF_11)). Midwives use V-MIS during their normal consultations with pregnant smokers or plan a separate consultation to provide smoking-cessation counselling.

Perinatal mortality in the Netherlands used to be higher than in other European countries so there was room for improvement in perinatal care, specifically in smoking behaviour ([12](#_ENREF_12)).

STIVORO and professional midwifery organisation collaborated in the provision of support to midwifery practices to improve smoking-cessation counselling. STIVORO has a programme to reduce second-hand smoke, which also includes the reduction of pregnant smokers. They developed the V-MIS in cooperation with a scientific institute and offer training to midwives about how to use V-MIS in their practice. Self-help materials are also provided by STIVORO.

The Dutch professional midwifery organisation represents the interests of midwives in the Netherlands in a powerful way. Besides their national office, in each region of the Netherlands they have a local network of midwives, called a circle group. In these circle groups, they discuss all aspects of midwifery care in the Netherlands and the national office can provide input to these meetings. All chairs of these circle groups meet regularly with the national office. At these chair meetings other stakeholders may also introduce relevant topics. The chairs then pass the information to the other midwives in their region.

**References**

5. de Vries H, Bakker M, Mullen PD, et al. The effects of smoking cessation counseling by midwives on Dutch pregnant women and their partners. Patient Educ Couns. 2006;63:177–87.

8. Royal Dutch Organisation of Midwives. In: de Geus M, Cadée F, editors. Midwifery in the Netherlands 2012. Utrecht: ; 2012.

9. Wiegers TA, Warmelink JC, Spelten ER, et al. Work and workload of Dutch primary care midwives in 2010. Midwifery. 2013.

10. Dutch Healthcare Authority (Nederlandse Zorgautoriteit). Midwivery (Verloskunde). Available at:

http://www.nza.nl/zorgonderwerpen/zorgonderwerpen/verloskunde/. Accessed 22 July 2014.

11. Prochaska JO, DiClemente CC. Stages and processes of self-change of smoking: toward an integrative model of change. J Consult Clin Psychol. 1983;51:390–5.

12. Mohangoo AD, Buitendijk SE, Hukkelhoven CWPM, et al. High perinatal mortality in the Netherlands compared to other European countries: Peristat II study (Hoge perinatale sterfte in Nederland vergeleken met andere Europese landen: de Peristat-II-studie). Ned Tijdschr Geneeskd.

2008;152:2718–27. Retrieved from http://www.ntvg.nl/publicatie/Hoge-perinatale-sterfte-Nederland-vergeleken-met-andere-Europese-landen-de-Peristat-II-studie
